# Over Pressure in Children Causing Brain Mischief

**Published:** 1892-10

**Authors:** J. A. Diggle

**Affiliations:** 1 Dane Street, Rochdale; London


### OVER-PRESSURE IN CHILDREN, CAUSING BRAIN MISCHIEF.

BY J. A. DIGGLE, L. S. A., LONDON.

The following cases show the inadvisability of attempting to force children forward in schools without sufficiently considering their different individual capacity for learning.

It is, I am afraid, much too common a cause of children's ailments nowadays, and has not been quite enough considered, I think, by parents and teachers. In the ordinary Board School, as at present constituted, every child in each standard must be pushed on, *pari passu*, with all the others, so as to get all, if possible, passed at the examination next ensuing, into the standard above. In the first case here noted, the fault, *fons et origo mali*, was with the parents in sending such a young child to school at all, but as both parents were factory workers, and there was only a slightly older boy besides, the child went to school with him.

Both cases were very similar in the outset, but the first was the most severe, and in both I thought at first the illness was enteric fever, the more so as being next door neighbors and residing on the banks of the river, which is very foul and much polluted, and on Sundays, when the water is low, great banks of festering abominations are exposed.

Case I. Alf C., a sharp and more than usually intelligent little boy of only four years and eight months, was seized on April 3d, at breakfast time, with sickness and pain in the head. He had been attending school for six months, and being naturally quick, as I have said, he had been encouraged to learn, and had already reached the final class in the infant department, and would have been put into the general school but for his age, which forbade it.

When I saw him at 11 A. M., he was in bed, slightly flushed, head very hot, and temperature 99.2 degs. Tongue rather foul. Complains of pain in the head, and avoids light. No further vomiting since breakfast. Gave him a mixture of potass. citrat. and tinct. aconiti and calomel, gr. j., with sugar.

April 4th. Passed a bad night, rambling and talking about school. Tongue rather cleaner. Temperature 99.4 degs. Milk diet. To continue mixture. Night temperature same as morning. Added k. br. gr. ij., aa dose to medicine.

5th. Night passed much the same as last. Lies very quiet and still, but easily roused, and then quite conscious. Temperature 100.2 degs. Thirsty. Tongue furred but moist. No pain in abdomen. Stool natural. Ordered antipyrin, grs. v. every three hours. Temperature at night, 100 degs. Been delirious all afternoon. Ordered ice-bladder to head.

6th. Rather better this morning. No diarrhœa. No spots on abdomen. Head, however, very hot, mother having taking off ice-bag at 4 A. M., as child slept. To be replaced. To have five mins. bromidia (Battle) every two hours. Temperature, 100.2 degs.; night temperature same.

7th. Much better. Fairly good night. Slept four hours; tweve midnight to four A. M. Playing with toys on bed when I saw him. Temperature, 99.2 degs. Tongue cleaner. To continue bromidia mixture.

8th. Not quite so well. Ice-bag again neglected, to my vexation. To be continued, as also mixture.

9th. Much better. Sitting up playing. Temperature, 99 degs. Ice-bag discontinued. Same mixture.

10th. Improving fast. Not much appetite. Quin., gr. 1-2, s. t. d.

11th. Up and dressed. Still improving. No headache or pain. Temperature normal. With the exception of a slight cough, all went on well until 14th, when I discontinued visiting.

The good effect of the ice and bromidia was very quickly apparent in this case, as also in the next.

Case II. John B., a strong, sturdy, rough lad of just over 7 years of age, was a contrast to A. C., in that he was anything but fond of lessons, and rather dull in all subjects except drawing, in which he excelled.

He had failed last year in the examination, and in consequence, his teacher had been urgent as to the necessity of his passing this time, and had been, perhaps, rather too sharp on the lad.

Just a fortnight before the examination, on April 21st, he was seized also at breakfast time, with vomiting and pain in the head.

When I saw him in the forenoon, he was lying on a bed-chair, very drowsy, and resenting being roused. Had vomited every few minutes since breakfast, at which he had only drunk a cupful of coffee. Head very hot. Pupils contracted ; buries his face in the pillow. Temperature, 100 degrees.

Ordered cold water cloths to head until ice-bladder could be got, and a potass. citrat. mixture.

April 22d. Sickness relieved. No delirium, but wanders when roused, and talks of his play. Ice to head. Bromidia, m. v., every two hours. Night much the same. Temperature, 100 degrees.

23d. Much better. More easily roused, and sensible, though when left to himself, lies quiet for hours. Temperature, 99 degs. To continue bromidia and ice.

24th. Better. Sitting up in bed with his drawing-book. No dullness or drowsiness. Complains of no pain at all. Appetite not good. Quin., gr. 1-2, t. d. s.

25th. Appetite improved. No bad symptoms. Temperature normal. Playing about the bed-room.

26th. Ceased visiting. Boy going on well.

It seems curious to me that two lads of opposite temperaments should be so similarly affected. One sharp and intelligent, though very young and not compelled to learn ; the other older and duller, probably harassed by his teacher, and yet both develop almost the same symptoms. I may say that the younger child, a fortnight after I ceased seeing him, had a regular hysterical fit because his mother would not allow him to go to school with his brother, and was in a state of collapse, cold and pale, for two or three hours after.

The rapid improvement under the ice and bromidia treatment was very gratifying, and I have found bromidia very useful in such cases, and a reliable hypnotic whenever I have required to prescribe such a medicine.

1 Dane Street, Rochdale.—*The Hospital Gazette.*

